# Characteristics of Headache in Children Presenting to Ophthalmology Services in a Tertiary Care Center of South India

**DOI:** 10.7759/cureus.21805

**Published:** 2022-02-01

**Authors:** Joe Christopher, Yamini Priya, Vivek Bhat, GRK Sarma

**Affiliations:** 1 Ophthalmology, St. John's Medical College, Bangalore, IND; 2 Neurology, St. John's Medical College, Bangalore, IND

**Keywords:** asthenopia, migraine, secondary headache, primary headache, pediatric headache, refractive error

## Abstract

Introduction: Headache is a common cause of disability worldwide and can disrupt the education and social life of children. Children regularly present to ophthalmologists with headache. So, we aimed to describe the characteristics of headache in children presenting to the ophthalmology outpatient department (OPD) in our center.

Methodology: We conducted this cross-sectional, prospective study in Bangalore, India. We included all children aged 5-18 years, presenting with headache to the ophthalmology OPD, from September 2018 to September 2020, and excluded nonverbal children, those with prior head trauma, diagnosed psychiatric illnesses, or epilepsy. We provided each child with a headache questionnaire, following which they received a detailed ophthalmologic evaluation. We performed relevant descriptive and inferential analyses.

Results: We included 311 children, with a mean age of 11.1 years. Sixty-eight percent were males. Fifty-one percent reported holocranial headache, and 28% reported frontal headache. Sixty-nine percent reported screen time of ≥2 hours/day. The most common refractive error (RE) was myopia, seen in 48%. The most common type of headache was headache associated with refractive errors (HARE), seen in 64%, followed by migraine, in 19%. Children with HARE were more likely to be males, have daily screen time of >2 hours/day, or have myopia. Their headache was more likely to be for >1 month, or have frontal localization. Children with headache due to other causes were more likely to be adolescents.

Discussion: We found that almost two-thirds of children presenting to our ophthalmology OPD had HARE. Our findings support the association of REs with headache. Children with HARE had a longer history and predominantly frontal localization. Further, they reported longer screen time, a significant finding in today’s world. Ophthalmologists must be aware of the various etiologies of headache and ensure that each child with headache receives a full ophthalmologic evaluation.

## Introduction

Headache is a leading, yet under-recognized cause of disability worldwide [1]. The overall one-year prevalence of headache in India is 64% [1]. School-based cross-sectional studies worldwide have reported a headache prevalence of about 20% in younger children, and up to 88% in adolescents [2,3]. Children with headache have a lower health-related quality of life, and suffer a more significant impact on their education, due to school absenteeism and poor scholastic performance [2].

Ophthalmologists regularly see children with complaints of headache [4]. These complaints may or may not be genuinely ophthalmologic in origin. The International Classification of Headache Disorders, 3rd Edition (ICHD-3) has four broad categories for headache due to ophthalmologic causes - ‘headache attributed to acute angle-closure glaucoma’, ‘trochlear headache’, ‘headache attributed to ocular inflammatory disorder’, and ‘headache attributed to refractive error’ [5]. The latter has become particularly relevant given the ubiquity of electronic devices and the associated rise in eye strain [6].

The potential of headache for significant morbidity necessitates thorough evaluation and appropriate treatment. So, we aimed to describe the characteristics of headache in children presenting to the ophthalmology outpatient department (OPD) at a tertiary care center in South India. We also aimed to describe the characteristics of headache associated with refractive errors (HARE).

## Materials and methods

We conducted this cross-sectional, prospective study at a tertiary care center in Bangalore, India. We included all children, aged 5-18 years, presenting with headache to the ophthalmology OPD from September 2018 to September 2020. We excluded those with prior history of head trauma, those with diagnosed psychiatric illnesses, those with epilepsy, and nonverbal children.

After taking informed consent from the guardian and assent from the child, we provided a headache questionnaire. The questionnaire consisted of 22 questions regarding headache characteristics and past and family history of headache (Appendix A). Additionally, it covered the child’s daily electronic gadget use and screen time. 

Following this, each child received a detailed ophthalmologic evaluation, including visual acuity, ocular alignment, a slit lamp assessment, and fundus examination. We defined visual impairment as visual acuity <6/18, as per the International Classification of Diseases, 10th Edition [7]. Relevant imaging was performed when necessary, and those children requiring further assessment by other specialists received it.

We entered data on Microsoft Excel and performed relevant analyses on SPSS version 21 (IBM Corp, Armonk, NY). We calculated percentages, means, and standard deviations for descriptive statistics. For categorical data, we used Pearson’s chi-squared test, and Fischer's exact test, where appropriate, with the statistical significance level set at 0.05.

## Results

We included 311 children, of which 210 (67.5%) were males. We excluded 25 children, of whom 10 had prior head trauma, eight had epilepsy, five had coexisting psychiatric diagnoses, and two were nonverbal. The mean (standard deviation [SD]) age was 11.1 (2.8) years. Two hundred and fourteen (68.8%) belonged to the adolescent age group (10-18 years).

Characteristics of headache

The headache duration varied from one week to 12 months among our subjects. Forty-one (13.2%) reported a duration of <1 month, 230 (74%) of 1-6 months, and 40 (6.4%) of >6 months.

One hundred and fifty-eight (50.8%) children localized the headache as holocranial, 86 (27.7%) as frontal, and 67 (21.5%) children described nonspecific localization.

Two hundred and fifteen (69.1%) reported a screen time of >2 hours/day. Two hundred and forty-four (78.5%) children had no history of headache in the family. Twenty-two (7.1%) reported headache in one generation, and 31 (9.9%) reported headache in two generations of family. Fourteen (0.5%) reported headache in siblings.

Ophthalmologic characteristics

One hundred and ten (35.4%) children were visually impaired, with visual acuity of <6/18. Further evaluation revealed some degree of myopia in 150 (48.2%) children (100 males, 50 females), hypermetropia in 50 (16.1%) children (50 males), and astigmatism in 30 (9.6%) children (20 males, 10 females).

Fundoscopic evaluation revealed papilledema in one child and hypertensive retinopathy in two children. All other children had normal findings. Slit lamp evaluation revealed no abnormalities.

On formal evaluation and the application of ICHD-3 criteria, the most common type of headache was HARE, seen in 198 (63.7%). Eighty-two (26.4%) were diagnosed with primary headache - either migraine or tension headache. Other secondary causes included sinusitis (17 children), hypertension (two children), and cerebral sinus venous thrombosis (one child) (Table 1).

**Table 1:** Etiology of headache in 311 children presenting to the ophthalmology OPD OPD: outpatient department

Headache etiology	Male	Female	Total
Refractive error	122	76	198 (63.7%)
Migraine	40	20	60 (19.3%)
Tension headache	12	10	22 (7.1%)
Others	19	12	31 (9.9%)

HARE versus other causes of headache

One hundred and ninety-eight (63.7%) children were diagnosed with HARE. One hundred and forty-eight (74.7%) were male. The mean (SD) age was 11.3 (3.0) years, with 138 (69.7%) children being adolescents.

All these children reported headache that was aggravated by visual activities, such as watching television, or using a mobile, and relief on halting device exposure. One hundred and seventy-eight (89.9%) reported a duration of headache that was >1 month long.

One hundred and forty-nine (75.3%) children had myopia, 29 (14.6%) had hypermetropia, and 20 (10.1%) had astigmatism. Among those with myopia, the most common location of headache was frontal, reported by 63 (42.3%), followed by holocranial (40.3%). Holocranial headache was most commonly reported in both those with hypermetropia (21-72.4%) and those with astigmatism (14-70%). None of those with hypermetropia or astigmatism reported frontal headache.

The mean (SD) duration of headache in those with myopia was 3.3 (1.7) months, while the mean (SD) durations for hypermetropia and astigmatism were 3.7 (0.9) and 3.4 (2.6) months, respectively. One hundred and twenty-seven (85.2%) of those with myopia reported headache of >1-month duration, while all of those with hypermetropia, and 10 (50%) of those with astigmatism reported similar durations. The vast majority of those with myopia or hypermetropia reported screen times of >2 hours/day, compared to only one child (5%) among those with astigmatism. Only a small minority reported a family history of headache. On univariate analysis, children with myopia were more likely to report frontal location of headache, and a shorter duration of headache compared to those with hypermetropia (Table 2).

**Table 2:** Characteristics of headache in those with myopia compared to those with hypermetropia

	Myopia (n = 149)	Hypermetropia (n = 29)	p-Value
Frontal headache	63 (42.3%)	0	<0.00001
< 1-month duration of headache	29 (19.5%)	0	0.005
>2-hour screen time	127 (85.2%)	23 (79.3%)	0.42
Family history of headache	16 (10.7%)	1 (3.4%)	0.31

On univariate analysis, children with spherical REs (myopia or hypermetropia) were more likely to report frontal headache, while those with astigmatism were more likely to report holocranial headache. Further, those with astigmatism were more likely to have a shorter duration of headache, and less likely to report longer screen times (Table 3).

**Table 3:** Characteristics of headache in those with spherical REs (myopia or hypermetropia) compared to those with astigmatism RE: refractive error

	Spherical RE (n = 178)	Astigmatism (n = 20)	p-Value
Frontal headache	63 (35,4%)	0	0.0005
Holocranial headache	81 (45.5%)	14 (70.0%)	0.037
<1-month duration of headache	29 (16.3%)	10 (50.0%)	0.0003
>2 hours screen time	150 (8.4%)	1 (5.0%)	<0.00001
Family history of headache	17 (9.6%)	1 (5.0%)	0.45

On chi-square, children with HARE were more likely to be males, have daily screen time exposure of 2 hours/day, or have myopia. They were more likely to have headache of >1-month duration and have a frontal headache. Children with headache due to other causes were more likely to be of the adolescent age group and were more likely to have a shorter duration (<1 month) of headache. There was no significant difference regarding the family history or the presence of hypermetropia or astigmatism (Table 4).

**Table 4:** Characteristics of HARE compared to other causes of headache HARE: headache associated with refractive errors

Characteristic	HARE (n = 198)	Other causes (n = 113)	p-Value
Male	148 (74.7%)	62 (54.9%)	0.0003
Adolescent	88 (44.4%)	73 (64.6%)	0.0006
Duration <1 month	20 (10.1%)	21 (18.6%)	0.0334
Frontal location	63 (31.8%)	23 (20.4%)	0.0297
Screen time >2 hours/day	151 (76.3%)	64 (56.6%)	0.0003
Family history of headache	28 (14.1%)	25 (22.1%)	0.07
Myopia	149 (75.3%)	1 (0.1%)	<0.00001
Hypermetropia	29 (14.6%)	21 (18.6%)	0.36
Astigmatism	20 (10.1%)	10 (8.8%)	0.72

## Discussion

We found that almost two-thirds of children presenting to our ophthalmology OPD had HARE. This proportion is similar to that found by Rao et al., from north India, who reported refractive errors (REs) in 58.8% of children presenting with headache to their ophthalmology services [8]. This is, however, a much higher proportion than studies including children in general pediatric or neurology services - Talebian et al. found that migraine was the most common cause of headache, seen in 58.8% of their cohort of general pediatric patients [9], and Dotan et al. reported that uncorrected REs were the cause in only 1.7% of hospitalized children with headache [10]. We believe that our findings reflect selection bias inherent to our patient population.

Migraine was the second most common cause of headache in our sample, seen in almost 20%. This is similar to the findings of Rao et al. [8], who studied Indian children, and Xavier et al., who surveyed Brazilian adolescents [11]. Tension headache was diagnosed in 7%. This is a much greater proportion than that found by Rao et al. [8] and Talebian et al. [9], but lower than that found by Xavier et al. [11]. Reasons for this discordance could be related to the psychosocial environment of the study population.

The prevalence of headache in the Indian population gradually rises with age, peaking at around 45 years. Further, it has been shown that females suffered more disability than males [12]. In our sample, the vast majority were adolescents, with the minority being younger children. However, unlike the India-wide pattern, there was a strong male predominance, both in the total sample and among those with HARE. Rao et al. [8] and Dandona et al. [13] had similar findings in their samples from North India. However, we believe this is simply due to selection bias.

The causative role of REs in headaches has often been brought into question [14]. Some hypothesize that this is merely a mistaken association due to the high prevalence of both REs and headache. However, numerous other authors have reported headache as the presenting symptoms of REs, ranging from 15% to 70% of such children [4]. Daiban et al. found headache to be the presenting complaint in over half their patients with astigmatism, and almost 25% of those with myopia or hypermetropia [15]. Akinci et al. demonstrated a higher incidence of headache in children with astigmatism [16], and Hendricks et al. demonstrated a statistically significant association between both spherical and cylindrical REs, and headaches in children [17]. Additionally, Rao et al. found that headache had good predictive value of REs in children [8].

For a diagnosis of ‘HARE’, in addition to the presence of an uncorrected/miscorrected RE and the lack of another headache subtype diagnosis, ICHD-3 requires at least two of the following four criteria to be met: the headache has developed and/or significantly worsened in temporal relation to the onset or worsening of the RE(s); it has significantly improved after correction of the RE(s); it is aggravated by prolonged visual tasks at an angle or distance at which vision is impaired; or it significantly improves when the visual task is discontinued [5].

Due to lack of follow-up data in our cohort, almost all children were diagnosed with HARE on the basis of the relationship of headache with visual tasks. This is supported by the statistically significant association of HARE with a history of greater screen times. Further, most of these children had a longer history of headache, which we hypothesize was related to the onset or worsening of REs.

Lajmi et al. described HARE as chronic, progressive, daily, and greater toward the end of the day while decreasing on vacation days. Further, they reported that HARE was usually localized to the frontal and orbital areas [4]. Our findings echo theirs. We found that children with HARE were more likely to have frontal headache compared to children with headache due to other causes. They also tended to have a longer history of complaints (>1 month). Family history did not have a significant association. However, these associations were limited to spherical REs - myopia and hypermetropia - and did not apply to astigmatism. Potential reasons for the differences in headache characteristics among those with astigmatism compared to spherical REs need to be explored. The differences in headache seen in myopia and hypermetropia - frontal headache being more common in myopia, for example - are also worth exploring.

HARE demonstrated a statistically significant association with the presence of myopia, the most frequent RE, rather than hypermetropia or astigmatism in our cohort. Rao et al. also reported myopia to be the most common RE in their cohort [8]. However, our findings contrast with those of Lajmi et al., who reported an association of HARE with moderate hypermetropia and astigmatism [4], and Akinci et al., who reported astigmatism to be the most common RE in their cohort of children with headache [16].

Hypothesized mechanisms for HARE include sustained accommodative effort in those with hypermetropia and squinting, by scalp and periorbital muscle contraction, to create a pinhole effect in myopia [18]. Prolonged exposure to screens and/or prolonged reading has been demonstrated to be a risk factor, not just for HARE but for primary headache as well [4,11]. This is further relevant given the still-ongoing COVID-19 pandemic, where most children have been forced to transition to online schooling. A recent survey reported that about half of the surveyed children suffered symptoms of headache and/or eye strain due to increased screen time during this period [6].

Other secondary causes of headache included sinusitis, seen in 9% of our sample. Uncommon causes included secondary hypertension in two children and cortical sinus venous thrombosis (CSVT) in one child. While these are uncommon, our experience supports the need for detailed evaluation in children presenting with headache. This will not only identify the underlying cause but also alleviate parental anxiety.

Limitations of our study include the lack of an age-matched control of children without headache. We did not evaluate psychosocial factors that may have contributed to presenting complaints. We did not formally collect data on the intensity of the headache. On history, almost all had mild to moderate intensity of headache. Only one child, who was later diagnosed with CSVT, had severe headache, and had an acute presentation. Finally, we do not have long-term follow-up data to assess the progression or resolution of each child’s headache. Our patients come from all over India, and many belong to the lower socioeconomic strata. A significant proportion of our data was collected during the first wave of the COVID-19 pandemic, when India had a strict lockdown protocol. All these reasons contributed to difficulty in collecting follow-up data and contacting the patients.

## Conclusions

We found that HARE was the most common cause of headache among children presenting to the ophthalmology OPD in our center. Children with HARE most commonly had myopia. They tended to have frontal headaches, and a longer history of headache compared to other causes.

Children with headache often make their first clinical encounter with the ophthalmologist. Ophthalmologists must ensure that every child with headache receives a thorough evaluation, including, but not limited to, a complete ophthalmologic evaluation. This is important not just to potentially identify rare etiologies and provide appropriate treatment, but also to relieve health-related anxiety.

## Figures and Tables

**Figure 1:**
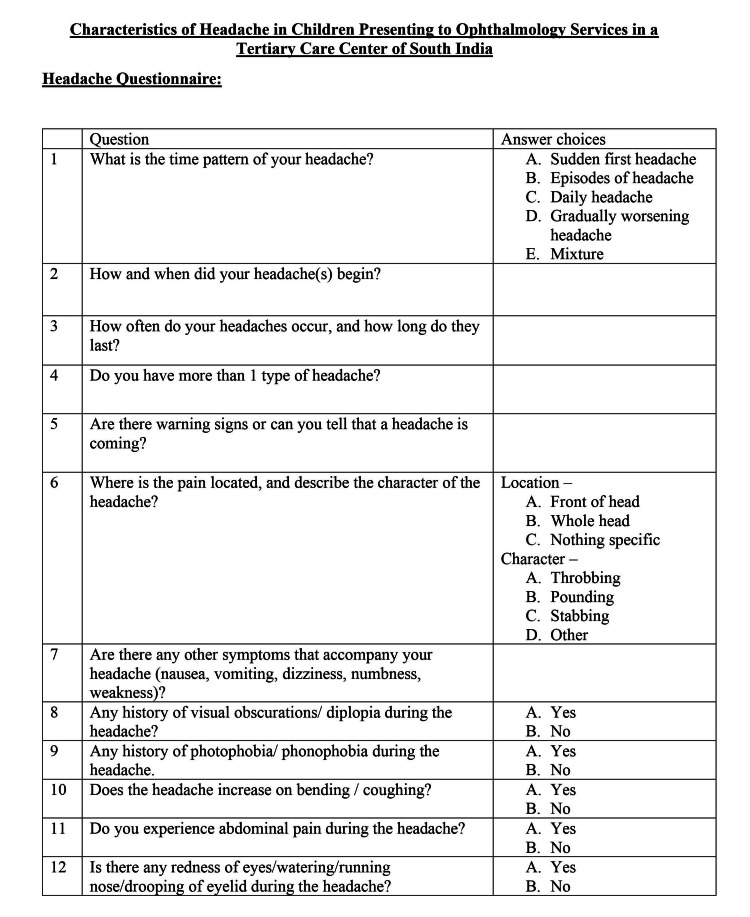
Headache questionnaire—front Blank cells indicate that the participants were to respond freely.

**Figure 2:**
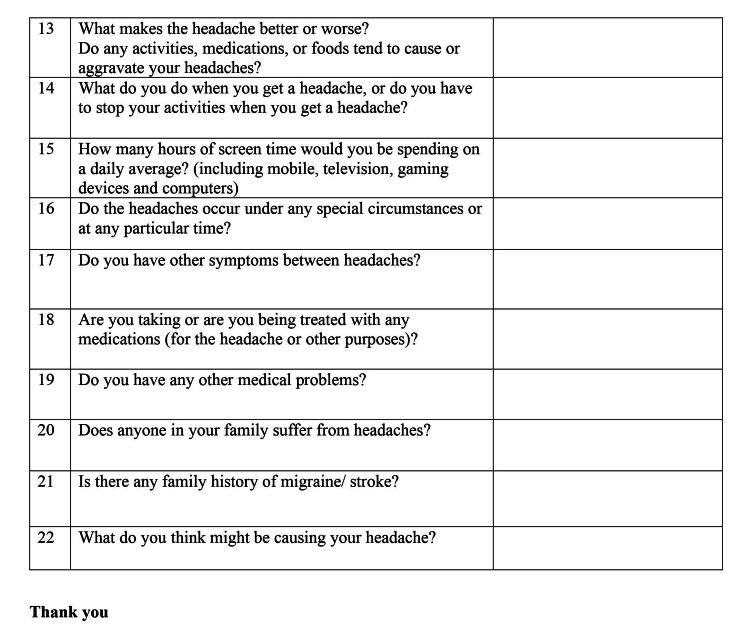
Headache questionnaire—back Blank cells indicate that the participants were to respond freely.

## Appendices

Appendix A 
